# Management of the Airway for Transoral Robotic Supraglottic Partial Laryngectomy

**DOI:** 10.3389/fonc.2018.00312

**Published:** 2018-08-14

**Authors:** Vanessa C. Stubbs, Karthik Rajasekaran, Adam R. Gigliotti, Ahmad F. Mahmoud, Robert M. Brody, Jason G. Newman, Christopher H. Rassekh, Gregory S. Weinstein

**Affiliations:** ^1^Department of Otorhinolaryngology: Head and Neck Surgery, University of Pennsylvania, Philadelphia, PA, United States; ^2^Department of Otorhinolaryngology: Head and Neck Surgery, Medical University of South Carolina, Charleston, SC, United States

**Keywords:** transoral robotic surgery, supraglottic partial laryngectomy, organ preservation surgery, airway, tracheostomy

## Abstract

**Introduction:** Over the last several decades, transoral resection techniques for treatment of supraglottic lesions have become increasingly favored to reduce the need for either open transcervical resection or primary chemoradiation. Transoral robotic surgery (TORS) offers advantages in visualization, dissection control, and access to remove bulky tumors en bloc. However, the management of the airway for these cases tends to vary, without clear guidelines as to when a tracheostomy is necessary.

**Materials and Methods:** A retrospective review of all patients who underwent transoral robotic supraglottic partial laryngectomy at a large academic center from May 2005 through December 2016 was performed. Airway management was examined, specifically as it pertains to whether a tracheostomy was performed at the time of surgery or otherwise. Demographic and tumor characteristics were also evaluated.

**Results:** Sixty-three patients were included. Forty (63%) were male, the average age at surgery was 63.6, and the majority (90.5%) underwent resection for squamous cell carcinoma of the supraglottis. Thirty-nine patients (62%) underwent the procedure with standard endotracheal intubation using a wire-reinforced tube. Of these, four patients required subsequent tracheostomy- 2 for laryngeal edema postoperatively, one for airway management during a postoperative bleeding event, and one for laryngeal edema following initiation of adjuvant chemoradiation. Twenty patients (32%) underwent tracheostomy at the time of transoral resection for airway management, 17 of whom were decannulated an average of 12.2 weeks following surgery. Those who underwent tracheostomy at the time of surgery had a higher percentage of tumors involving multiple supraglottic subsites (*p* = 0.031), 85 vs. 54% in the group who did not undergo tracheostomy. No difference in age, BMI, clinical T-stage, or clinical N stage was found between the two groups.

**Conclusion:** Performing a tracheostomy at the time of surgery should be considered for those patients with more extensive malignant disease (≥T2 tumors). While avoiding tracheostomy is often preferred by the patient, the maintenance of the patent airway peri-operatively should be first priority when considering airway management. Furthermore, as the majority of those patients receiving tracheostomy are decannulated within 4 months of surgery, the tracheostomy could be considered a short-term adjunct to the procedure.

## Introduction

Over the last few decades there have been several shifts in optimal treatment regimens for laryngeal cancer. While primary chemoradiation therapy has been utilized widely as an “organ preservation” option since the publication of the Veteran Affairs (VA) trial in 1991 to avoid disfigurement and morbidity associated with previous open surgical resections, this regimen has not been without its own toxicities (1, 2). With some series reporting a 24% incidence of persistent dysphagia, 10% incidence of long term enteral access for feeding, and overall poor quality of life, there has been a movement toward minimally invasive transoral surgical techniques (3, 4). Both transoral laser microsurgery (TLM) and transoral robotic (TORS) partial supraglottic laryngectomy have been introduced as surgical organ preservation options. TLM has been shown to have both equivalent oncologic outcomes to open resection and chemoradiation as well as decreased morbidity, shorter hospital stays, and superior functional outcomes (5–7). Due to limitations of TLM including exposure, ability to obtain en bloc resection and difficulty in resection of large bulky tumors, TORS supraglottic partial laryngectomy was introduced as an alternative methodology by Weinstein et al. in 2007 following good feasibility outcomes utilizing TORS in resection of oropharyngeal malignancy (8–10). In several feasibility studies and institutional series published, TORS partial laryngectomy has been considered a safe and oncologically sound treatment option (11–16). However, there is a lack of data in these studies regarding airway management and need for tracheostomy. Traditionally, tracheostomy has been indicated for several reasons in this setting: to provide exposure and additional operative space during surgery in patients with bulky tumors, to maintain a patent airway in those with postoperative laryngeal edema, and to protect the lower airway in the case of bleeding event postoperatively. In this study, we aim to evaluate the airway management technique in this institution's series of patients undergoing TORS supraglottic partial laryngectomy.

## Materials and methods

A retrospective review of all patients who underwent transoral robotic supraglottic partial laryngectomy at a large academic medical center from May 2005 through December 2016 was performed. Institutional Review Board approval was obtained for this retrospective study from the University of Pennsylvania Office of Clinical Research, and all subjects gave written informed consent. The electronic medical record system as well as archived paper medical records were utilized to collect patient demographic information including age, sex, weight, body mass index (BMI), past medical history and active medications, specifically anticoagulation/antiplatelet therapies, at the time of surgery; tumor information including tissue diagnosis, primary disease subsite within the supraglottic, involvement of multiple supraglottic subsites, TNM stage, pre-epiglottic, and paraglottic space involvement; and treatment information including margin status, management of the nodal disease, need for neck dissection, and need for adjuvant therapy.

Airway management information was also obtained including tracheostomy placement timing, date of decannulation if applicable, long term tracheostomy tube dependence, and reason for delayed tracheostomy placement if applicable.

The transoral robotic supraglottic partial laryngectomy procedure is a standardized procedure at our institution with bilateral supraglottic resection performed routinely for all tumors.

Neck dissection was the primary treatment for management of the neck in this patient cohort as determined by the surgeon in conjunction with the multidisciplinary tumor board. Adjuvant radiotherapy with or without chemotherapy was recommended for patients with pathology results indicating close or positive surgical margins for a tumor at the primary site, involvement of 2 or more lymph nodes, perineural or lymphovascular invasion, or extracapsular spread of nodal metastasis.

Two sample *t*-testing, two-sample test of proportions, two-sample Wilcoxon rank sum (Mann-Whitney), and Fisher exact testing was used to compare differences in characteristics between patients who underwent tracheostomy at the time of TORS and those who did not undergo tracheostomy. Multivariate regression analysis was used in order to assess the relationship between time to decannulation with adjuvant radiation and chemotherapy. Statistical analysis was performed using Stata (Stata Statistical Software: Release 15. College Station, TX: StataCorp LLC).

## Results

Sixty-three patients underwent transoral robotic partial laryngectomy at our institution between May 2005 and December 2016. There was a male predominance (63%), an average age at surgery of 63.6 years (range [20, 84], *SD* = 10.4) and an average follow-up time of 49.7 months (range [2.4, 126.9], *SD* = 34.8). The majority of patients (91%) underwent resection for squamous cell carcinoma. The other pathologies are listed in Table 1. The majority of lesions were primarily involving of the epiglottis (62.0%), followed by the aryepiglottic fold (14.3%), the arytenoid (6.3%), and the false cord (1.2%). Forty patients presented with multiple subsites involved (63.5%). Patients included with epithelial carcinomas had T1-T3 tumors, majority with T2 tumors (44.8%). Tumor data was unable to be obtained for five patients.

**Table 1:** Disease pathology of all included patients.

**Pathology**	***N* (%)**
Squamous cell carcinoma	57 (90.5%)
Paraganglioma	2 (3.2%)
Adenosquamous carcinoma	1 (1.6%)
Chondrosarcoma	1 (1.6%)
Mucoepidermoid carcinoma (low grade)	1 (1.6%)
Amyloidosis	1 (1.6%)

There were no intraoperative complications (0%). Two patients experienced temporary postoperative complications: one patient experienced postoperative bleeding requiring surgical intervention, and one patient experienced a myocardial infarction for which he underwent subsequent coronary bypass surgery. Length of hospitalization ranged from 2 to 37 days (median = 5, *SD* = 6.3).

Neck dissection was indicated and performed in 74.6% of patients, the majority of which were bilateral (78.7%). Timing of the neck dissection with regards to the TORS supraglottic partial laryngectomy resection varied: 74.5% of patients underwent neck dissection following TORS procedure (at a mean of 39 days following the procedure), 23.4% underwent neck dissection prior to TORS resection, and 2.1% had neck dissection performed at the time of the TORS resection. Thirty-two percent of patients underwent adjuvant radiotherapy and 25% underwent adjuvant chemotherapy.

With regards to airway management, 39 patients (62%) underwent the procedure with standard endotracheal intubation using a wire-reinforced tube and did not undergo tracheostomy at the time of the supraglottic partial laryngectomy. Of these, 4 patients required subsequent tracheostomy. One of these patients underwent tracheostomy for airway management during a second bleeding event post-operatively. This patient had previously been extubated without difficulty twice prior to placement of the tracheostomy tube 13 days following their initial resection and was subsequently successfully decannulated 5 weeks following without complication. Two patients underwent tracheostomy at 3 and 6 weeks following the TORS procedure due to laryngeal edema. The last patient underwent delayed tracheostomy (4 months after surgery) due to laryngeal edema following initiation of adjuvant chemoradiation.

Twenty patients (32%) underwent tracheostomy at the time of transoral resection for airway management, 17 of whom were subsequently decannulated an average of 12.2 weeks following surgery. From this group, 11 patients were decannulated an average of 38 days following surgery, six others had tracheostomy for more than 3 months (range 145–243 days), and 3 patients remained with a permanent tracheostomy. There were no airway complications postoperatively in this group of patients. An additional four patients who underwent the TORS supraglottic partial laryngectomy procedure had previously undergone tracheostomy prior to their procedure. Two of these patients had required tracheostomy on an emergent basis due to airway obstruction from their laryngeal tumor prior to consideration for surgical resection, while one patient had concomitant tonsillar squamous cell carcinoma and underwent tracheostomy previously. The final patient had recurrent chondrosarcoma of the arytenoid cartilage and had a long-standing tracheostomy prior to their procedure. All patients with prior tracheostomy were excluded from analysis regarding need for tracheostomy at the time of surgery.

Those who underwent tracheostomy at the time of surgery had no difference in age, sex, BMI, clinical T-stage, clinical N stage, or length of stay compared to those who did not undergo tracheostomy. However, there was a significant difference between the two groups when looking at tumor involvement of multiple subsites. Fifty-four percent of patients who did not undergo tracheostomy had tumors that involved multiple subsites, compared to 85% of patients who underwent tracheostomy (*p* = 0.031, 95% CI [−0.51, −0.06]). Patient characteristics of each group can be found in Table 2. There was also no difference in active use of anticoagulation or antiplatelet therapies (including warfarin, factor XA inhibitors such as rivaroxaban, clopidogrel, and full dose aspirin) between the two groups. Furthermore, no difference was found in history of pulmonary comorbidity.

**Table 2:** Characteristics of patients who underwent tracheostomy at the time of surgery compared to those in which tracheostomy was not performed at the time of resection.

**Characteristic**	**Tracheostomy at time of TORS**	**No Tracheostomy**	***P* value**
***N***	20	39	
**Age (mean**, ***SD*****)**	67.0 (8.4)	62.3 (11.3)	0.087
**Sex**			0.407
Male	14 (70.0%)	23 (59.0%)	
Female	6 (30.0%)	16 (41.0%)	
**Weight (kg)**	76.4	74.2	0.590
**BMI**	25.5	26.3	0.888
**History of pulmonary comorbidity**	3 (15.0%)	5 (12.8%)	0.758
**Active anticoagulation at time of surgery**	4 (20.0%)	5 (12.8%)	0.416
Warfarin	1	1	
Clopidogrel	2	3	
Aspirin (full strength)	1	1	
**Squamous cell carcinoma pathology**	20 (100%)	35 (89.7%)	
**Primary subsite**			0.806
Epiglottis	12 (60.0%)	24 (61.5%)	
Aryepiglottic Fold	3 (15.0%)	6 (15.4%)	
Arytenoid	2 (10.0%)	1 (2.6%)	
False vocal cord	0 (0%)	1 (2.6%)	
**Multiple subsites involved**	17 (85.0%)	21 (53.8%)	**0.031**
**T Stage**			0.212
T1	1	8	
T2	12	14	
T3	4	6	
Recurrence	3	3	
Unknown	0	5	
N/A (non-epithelial)	0	3	
**N Stage**			0.389
N0	17	24	
N1	0	3	
N2	3	7	
N3	0	0	
Unknown/NA	0	5	
**Length of stay (mean)**	5.3	7.4	0.512
**Neck Dissection Performed (% of patients)**	17 (85.0%)	29 (74.4%)	0.350
Unilateral (% of neck dissections performed)	4 (23.5%)	6 (20.7%)	0.822
Bilateral (% of neck dissections performed)	13 (76.5%)	23 (85.2%)	
Not performed	3	10	
**Timing of Neck Dissection**			0.070
Prior to TORS (% of neck dissections performed)	7 (41.2%)	4 (13.8%)	
Concurrent with TORS (% of neck dissections performed)	0 (0.0%)	1 (3.4%)	
After TORS (% of neck dissections performed)	10 (58.8%)	24 (82.8%)	
Not Performed	3	10	

*Statistically significant values with P < 0.05 in bold*.

In those who underwent tracheostomy at the time of TORS resection, 58.8% of patients had neck dissection following their surgery while 41.2% of patient had neck dissection performed previously. In the patients that did not receive tracheostomy, 82.8% of patients underwent delayed neck dissection while 13.8% of patients had neck dissections preformed previously and 3.4% had neck dissection at the time of TORS. While, a greater proportion of patients who did not receive tracheostomy had delayed neck dissections, this only trended toward significance (*p* = 0.070). Neck dissection data can be seen in Table 2.

For the patients who underwent tracheostomy during treatment, there was a significant difference in time to decannulation with regards to adjuvant therapy. For those who received adjuvant radiotherapy, there was a positive correlation with time to decannulation, with the average time to decannulation equal to 16 weeks compared to 14 weeks for those who did not receive radiation therapy (*p* = 0.039). Adjuvant chemotherapy had a negative correlation with time to decannulation with the average time to decannulation at 10 weeks compared to 17 weeks for those who did receive chemotherapy (*p* = 0.023).

**Figure 1:**
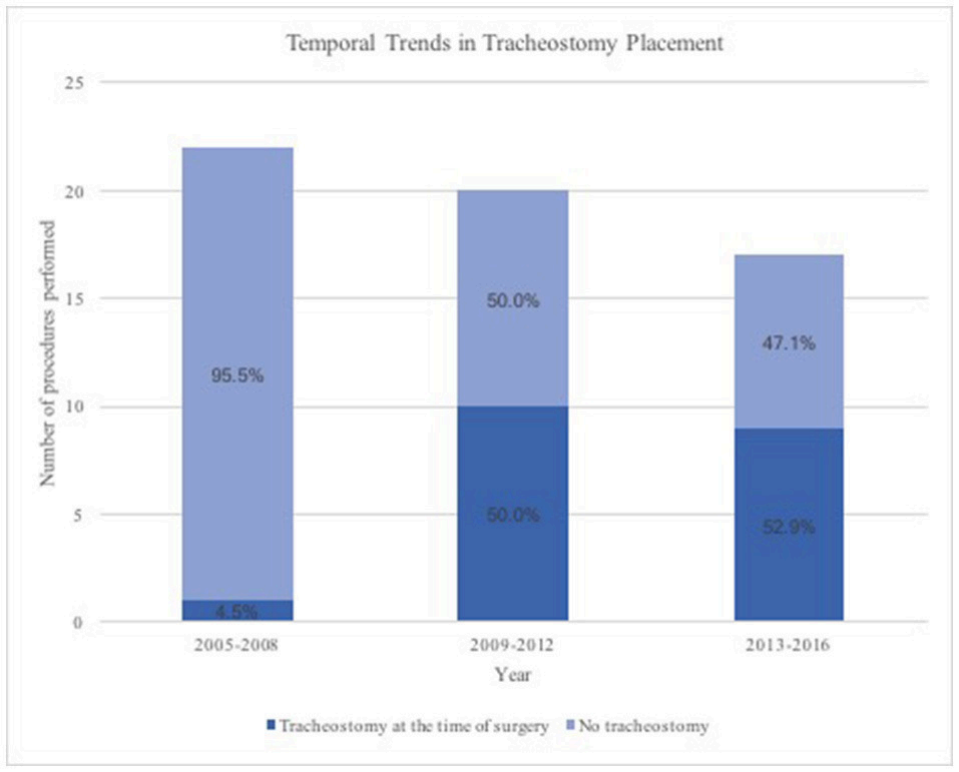
Temporal Trends in Tracheostomy placement during transoral robotic supraglottic partial laryngectomy.

A trend toward tracheostomy at the time of surgery was found over the course of the study period (Figure 1). From the years 2005 through 2008, 22 patients underwent TORS supraglottic partial laryngectomy and only 1 patient (4.5%) underwent tracheostomy at the time of surgery. Of the 20 patients who underwent the procedure from 2009 through 2012, 10 patients (50%) underwent tracheostomy at the time of surgery. From 2013 through 2016, 9 patients (52.9%) underwent tracheostomy at the time of TORS procedure out of a total of 17 patients. A similar trend in tumor size was not found during this time period. From 2005 through 2008, 21% of patients presented with T1 disease, with 79% having > = T2 disease. While only 6% of patients from 2009 through 2012 had T1 disease (94% with > = T2 disease), 31% presented with T1 disease (69% with > = T2 disease) during the years 2013 through 2016.

## Discussion

In this retrospective study, we examined airway management, specifically need for tracheostomy, during TORS supraglottic partial laryngectomy over a 16-year period. While several previously published institutional studies have commented on airway management, they have primarily focused on the feasibility of performing the procedure rather than on how the airway was managed. Mendelsohn et al. published their experience with TORS supraglottic partial laryngectomy in 2013. The study examined the cases of 18 patients, all of whom completed the procedure with orotracheal intubation and were kept intubated until post-operative day one (11). Similarly, in their series of 13 patients in 2013, Ozer et al. noted that no patient underwent tracheostomy at the time of surgery and all patients were extubated immediately post-operatively; however, one patient required delayed tracheostomy (15). Park et al. also published a feasibility study in which 16 patients were included, all of whom underwent temporary tracheostomy at the time of surgery with a mean time of decannulation of 11 days (12). Given that the dataset in this study examines both cases in which the airway was managed by both intubation only and also tracheostomy at the time of surgery, the outcomes of each could be evaluated.

Furthermore in 2015, Razafindranaly et al. published a multicenter retrospective study examining outcomes following transoral robotic supraglottic partial laryngectomy including 84 patients (16). Of these, 20 patients (24%) underwent tracheostomy, 12 of whom underwent simultaneous tracheostomy during the time of surgery due to high risk for laryngeal edema postoperatively and 8 who underwent tracheostomy following resection due to dyspnea postoperatively. The overall tracheostomy rate of 24% of this study was slightly less than what we found in the present study during which 38% underwent tracheostomy; however, a larger proportion of those receiving tracheostomy in our study underwent tracheostomy during the resection as compared to post-operatively in the setting of dyspnea (83 vs. 60%). As the patient T stages were similar between the two studies with majority being T2 lesions, this may reflect a lower threshold of our institution to prophylactically perform tracheostomy. Additionally, while the previous study discusses perioperative airway management, it did not mention which characteristics were taken into account in determining risk for postoperative laryngeal edema. The aim of the current study is to expand on those characteristics which may put patients at higher risk for need for tracheostomy.

Tumor involvement of multiple subsites was noted to be significantly different between the two groups, with an increased percentage of multiple subsite involvement in the tracheostomy group. Although no significant difference was found in clinical T stage as a whole between the groups, there was a difference when looking at proportions of T1 compared to ≥T2 tumors. This finding suggests that the presence of a tumor involving multiple subsites (≥T2) could be a factor in consideration of tracheostomy at the time of surgery. Given that the extent of resection during transoral supraglottic partial laryngectomy at our institution is standardized, ability to gain exposure and additional operative space was a factor in decision for tracheostomy for bulkier ≥T2 tumors and likely contributes to this finding.

It was also noted that neck dissection was more likely to be delayed in those patients who did not undergo tracheostomy at the time of TORS resection, though this only trended toward significance. Bilateral neck dissection is indicated in the majority of patients with supraglottic malignancy and carries the possibility of increased laryngeal edema if performed simultaneously with laryngeal resection. Therefore, this should be taken into account when considering airway management. In those patients without other risk factors undergoing the TORS procedure without tracheostomy, consideration should be given to staging the neck dissection.

While we found no difference in age, BMI, pulmonary comorbidities, active anticoagulation/antiplatelet medications, clinical T stage, clinical N stage, or length of stay between those who underwent tracheostomy at the time of surgery and those who did not, it should be noted that all patients who underwent tracheostomy had diagnoses of squamous cell carcinoma as opposed to other benign pathology. For each patient in this study, a priority was made to evaluate the optimal airway management for each patient on a case by case basis with emphasis placed on difficulty of exposure during intubation, extent of disease resection, and body habitus of the patient that may prevent easy reintubation.

For those who underwent tracheostomy, we found that time to decannulation was increased for those who underwent adjuvant radiotherapy. This correlation is likely due to acute side effects of radiation therapy including laryngeal edema. As 45% of the patients who underwent tracheostomy also underwent adjuvant radiotherapy, it is possible that this also increased the average time to decannulation that was longer in our study as compared to previous studies (12.2 weeks vs. 11 days in the study by Park et al.) (12). At our institution, a conservative approach was taken with regards to decannulation in order to avoid possible need for repeat tracheostomy at the time of adjuvant therapy when the procedure would have the potential to be more complicated given a previously operated neck or the possibility of requirement of urgent or emergent tracheostomy in a less controlled setting.

We also found a temporal trend at our institution toward tracheostomy over time. When looking at the same time periods, there was not a corresponding trend in tumor size over time that could explain this shift. This may be due to changing attitudes toward risk of airway complications postoperatively. While avoiding tracheostomy is often preferred by the patient, the maintenance of the patent airway peri-operatively should be first priority when considering airway management. Furthermore, it should be noted that the majority of those patients receiving tracheostomy were decannulated within 4 months of surgery. Therefore, the tracheostomy could be considered a short-term adjunct to the procedure.

This current study is limited by its retrospective nature. Although patient demographics and tumor characteristics were the main areas of examination, other factors that could not be corrected for may have played a part in airway decision making. Furthermore, given the temporal changes in practice over time, it is likely that surgeon preferences may also have contributed to changes in management. Although this study includes one of the largest published patient series, it nevertheless is still limited by sample size. Future large prospective studies would be needed to limit these biases; however, randomized trials would be difficult given patient strong preference against tracheostomy.

## Conclusion

While previous studies have examined the feasibility of performing TORS supraglottic partial laryngectomy, less emphasis has been placed on perioperative airway management. In the present study, we examined the management technique of the airway, whether tracheostomy was performed either at the time of surgery or otherwise, in 63 patients who underwent the procedure. A higher proportion of patients who underwent tracheostomy at the time of surgery had multiple subsite involvement of their tumors than those who underwent routine orotracheal intubation without tracheostomy placement. Those who did not undergo tracheostomy tended to have a staged neck dissection that could potentially limit laryngeal edema at the time of resection. Although we found no difference in age, BMI, pulmonary comorbidities, active anticoagulation/antiplatelet medications, or clinical N stage between the two groups, we did find a temporal trend toward tracheostomy at the time of surgery. This data supports the idea that each patient case should be evaluated individually, but also that tumor involvement of multiple supraglottic subsites (≥T2 tumors) may help to guide need for tracheostomy. While avoiding tracheostomy is often preferred by the patient, the maintenance of the patent airway perioperatively should be first priority when considering airway management. Ability to gain adequate access to the tumor for a complete oncologic resection may also play a part in decision making for tracheostomy. Given the results of this study, our institution is inclined to have a low threshold for temporary tracheostomy. If tracheostomy is performed, it is considered temporary and may prevent life threatening airway emergencies in the immediate postoperative period.

## Author contributions

VS: study design, preparation of manuscript, data collection, interpretation of data, and revision of manuscript. KR: study design, interpretation of data, and preparation of manuscript. AG, RB: study design and data collection. AM: study design, statistical analysis, and interpretation of data. JN, CR, GW: study design, interpretation of data, and revision of manuscript.

### Conflict of interest statement

The authors declare that the research was conducted in the absence of any commercial or financial relationships that could be construed as a potential conflict of interest.

## Abbreviations

TORS: transoral robotic surgery
TLM: transoral laser microsurgery
BMI: body mass index
SD: Standard Deviation.
